# Genome Sequences of 38 Bacteriophages Infecting Escherichia coli, Isolated from Raw Sewage

**DOI:** 10.1128/MRA.00909-20

**Published:** 2020-09-17

**Authors:** Troy C. Hinkley, Spencer Garing, Nick Clute-Reinig, Ethan Spencer, Paras Jain, Luis F. Alonzo, Anne-Laure M. Le Ny

**Affiliations:** a Intellectual Ventures Laboratory, Bellevue, Washington, USA; DOE Joint Genome Institute

## Abstract

Here, we report the complete genome sequences of 38 novel bacteriophages infecting Escherichia coli, isolated from a raw sewage source in Washington. Of these phages, 26 are under 100 kb, 11 are near 170 kb, and 1 352-kb jumbo phage was discovered.

## ANNOUNCEMENT

In this study, we isolated 38 Escherichia coli phages from raw effluent collected weekly at the King County wastewater treatment facility in Renton, Washington. Bacteriophages can be useful tools as antimicrobials or in diagnostics as reporter phages, for example. This work was undertaken as part of a larger study developing a field test kit to detect E. coli contamination in drinking water, with the goal to establish a large collection of phages specific to E. coli. To capture a diversity of phages, various strains of Escherichia coli were used as hosts (Table 1) according to methods described previously (1). Isolation hosts included strains selected from the Escherichia coli Reference (ECOR) Collection, obtained from the Thomas S. Whittam STEC Center at Michigan State University (2) (e.g., ECOR8), strains collected from various field sites (e.g., 04-01), and strains obtained from the E. coli Reference Center at Penn State School of Agriculture (e.g., PS1). Briefly, in a 150-ml flask, 25 ml raw sewage was mixed with 25 ml 2× LB broth and 500 μl of an overnight culture of E. coli host cells. After overnight incubation (37°C, 250 rpm), centrifugation (3,000 × *g*, 10 min), and filtration (0.22 μm), the supernatant yielded a phage lysate that was plaque purified three times on the original host strain to yield a homogeneous phage stock (3).

**TABLE 1:** Escherichia coli phage genometrics

Phage	Isolation host	Genome size (bp)	No. of ORF^*a*^	No of tRNAs	G+C content (%)	Mean coverage (×)	Clade	Type	Cluster	GenBank accession no.	SRA accession no.
TH06	ECOR8	77,678	121	1	42.1	78.2				MT446386	SRX8326066
TH09	ECOR29	167,656	268	11	35.5	57.2	*Myoviridae*	2		MT446387	SRX8326067
TH10	ECOR37	89,140	128	27	38.9	72.8	*Myoviridae*	1	7	MT446388	SRX8326078
TH11	ECOR38	45,305	64	0	54.4	51.8	*Siphoviridae*	1	5	MT446389	SRX8326089
TH12	ECOR49	169,545	269	2	37.6	75.4				MT446390	SRX8326098
TH13	ECOR50	45,377	65	0	54.4	56.9	*Siphoviridae*	1	5	MT446391	SRX8326099
TH15	ECOR68	171,591	280	2	40.3	55.5				MT446392	SRX8326100
TH16	04-01	40,612	52	0	50.1	62.1	*Podoviridae*	3		MT446393	SRX8326101
TH20	04-01	40,001	50	0	50.2	56.6	*Podoviridae*	3		MT446394	SRX8326102
TH21	13-08	52,087	85	0	47.3	59.2	*Siphoviridae*	1		MT446395	SRX8326103
TH22	PS1	170,090	291	2	40.3	74.2				MT446396	SRX8326068
TH23	PS2	52,433	72	0	44.0	54.9	*Myoviridae*	1		MT446397	SRX8326069
TH24	PS5	52,806	77	0	44.1	57.1	*Myoviridae*	1	7	MT446398	SRX8326070
TH25	PS6	51,729	72	0	44.0	56.5	*Myoviridae*	1	7	MT446399	SRX8326071
TH26	PS7	51,200	68	0	43.9	58.2	*Myoviridae*	1		MT446400	SRX8326072
TH27	PS8	52,344	86	0	46.6	56.7	*Siphoviridae*	1		MT446401	SRX8326073
TH28	PS10	55,824	80	0	44.1	47.8	*Myoviridae*	1	7	MT446402	SRX8326074
TH29	PS11	53,648	77	0	43.9	53.0	*Myoviridae*	1	7	MT446403	SRX8326075
TH30	PS13	51,754	86	0	47.2	53.3	*Siphoviridae*	1		MT446404	SRX8326076
TH32	PS17	69,233	101	0	46.0	51.1	*Myoviridae*	1	7	MT446405	SRX8326077
TH33	PS19	52,851	74	0	44.0	50.6	*Myoviridae*	1		MT446406	SRX8326079
TH34	PS21	77,944	124	1	42.3	51.4				MT446407	SRX8326080
TH35	PS22	69,093	99	0	46.0	62.3	*Myoviridae*	1	7	MT446408	SRX8326081
TH37	PS49	53,453	79	0	43.8	52.6	*Myoviridae*	1	7	MT446409	SRX8326082
TH38	PS50	81,552	131	1	42.2	52.0				MT446410	SRX8326083
TH40	PS53	171,726	289	2	40.3	54.1	*Myoviridae*	2		MT446411	SRX8326084
TH41	PS56	170,556	293	2	40.5	71.4				MT446412	SRX8326085
TH42	PS60	77,284	121	0	42.3	58.7				MT446413	SRX8326086
TH43	PS63	77,980	122	1	42.4	52.4				MT446414	SRX8326087
TH44	PS64	171,075	290	2	40.6	53.1				MT446415	SRX8326088
TH47	PS68	352,450	575	4	35.1	74.2	Jumbo			MT446416	SRX8326090
TH49	PS40	41,043	44	0	53.0	47.7	*Podoviridae*	3		MT446417	SRX8326091
TH50	PS40	41,778	47	0	53.1	52.1	*Podoviridae*	3		MT446418	SRX8326092
TH53	PS29	52,329	76	0	44.2	52.1	*Myoviridae*	1		MT446419	SRX8326093
TH54	PS37	170,988	292	2	40.4	52.2	*Myoviridae*	2		MT446420	SRX8326094
TH55	PS46	170,389	291	2	40.3	58.6				MT446421	SRX8326095
TH57	PS48	171,230	290	2	40.4	59.2	*Myoviridae*	2		MT446422	SRX8326096
TH58	PS71	170,748	275	2	37.5	55.7				MT446423	SRX8326097

a ORF, open reading frames.

Following propagation in liquid culture, phage lysates were treated with 2% SDS to liberate genomic DNA and purified using a Qiagen Genomic-tip 100/G kit according to the manufacturer’s specifications. Purified phage genomes were prepared for sequencing with the Nextera XT library prep kit and were sequenced on an Illumina MiSeq instrument, which generated 2 × 250-bp paired-end reads with an average insert size of between 350 and 450 bp. The mean coverage for each phage genome assembly is listed in Table 1. Adapter sequences were trimmed and poor-quality reads (<Q30) were filtered using Geneious Prime version 2019.1.1 before assembly with the Geneious Prime *de novo* assembler. Open reading frames were predicted via Glimmer gene model version 3.02 (4) and were classified into types and clusters using the Web server Virfam (5). ARAGORN version 1.2.38 (6) was used to predict tRNA genes, and PhageTerm was used to determine genomic termini (7). All tools were run with default parameters unless otherwise noted.

The genome sizes vary substantially in length from 40 kb (TH20) to over 350 kb (TH47). According to genome size, the phages can be fit into two groups (small and large), one averaging 59 kbp and the other averaging 171 kbp, with the 352-kbp phage serving as a jumbo phage outlier. Less variation was seen in G+C contents (from 35% to 54% in TH47 and TH13, respectively; Table 1) between the phages, with 32 of the 38 phages possessing less than 50% G+C content.

Most of the phages contain either 0, 1, or 2 tRNA genes; however; phages TH47, TH09, and TH10 contain 4, 11, and 27 tRNA genes, respectively (Table 1). According to the *in silico* morphologies determined by Virfam (5), most of the phages possess a siphoviral morphology. Phages with a podoviral morphology were found only among the small phages, while the large phages contained only myoviral and siphoviral morphologies.

### Data availability.

The sequence reads have been deposited in NCBI under the BioProject accession number PRJNA631765. The associated GenBank and Sequence Read Archive accession numbers are listed in Table 1.

## References

[1] Mattila S, Ruotsalainen P, Jalasvuori M 2015 On-demand isolation of bacteriophages against drug-resistant bacteria for personalized phage therapy. Front Microbiol 6:1–7. doi:10.3389/fmicb.2015.01271. 26617601 PMC4643220

[2] Ochman H, Selander RK 1984 Standard reference strains of Escherichia coli from natural populations. J Bacteriol 157:690–693. doi:10.1128/JB.157.2.690-693.1984. 6363394 PMC215307

[3] Kropinski AM, Mazzocco A, Waddell TE, Lingohr E, Johnson RP 2009 Enumeration of bacteriophages by double agar overlay plaque assay. Method Mol Biol 501:69–76. doi:10.1007/978-1-60327-164-6_7. 19066811

[4] Delcher AL, Bratke KA, Powers EC, Salzberg SL 2007 Identifying bacterial genes and endosymbiont DNA with Glimmer. Bioinformatics 23:673–679. doi:10.1093/bioinformatics/btm009. 17237039 PMC2387122

[5] Lopes A, Tavares P, Petit MA, Guérois R, Zinn-Justin S 2014 Automated classification of tailed bacteriophages according to their neck organization. BMC Genomics 15:1027–1017. doi:10.1186/1471-2164-15-1027. 25428721 PMC4362835

[6] Laslett D, Canback B 2004 ARAGORN, a program to detect tRNA genes and tmRNA genes in nucleotide sequences. Nucleic Acids Res 32:11–16. doi:10.1093/nar/gkh152. 14704338 PMC373265

[7] Garneau JR, Depardieu F, Fortier LC, Bikard D, Monot M 2017 PhageTerm: a tool for fast and accurate determination of phage termini and packaging mechanism using next-generation sequencing data. Sci Rep 7:1–10. doi:10.1038/s41598-017-07910-5. 28811656 PMC5557969

